# Rapidly Progressive Lupus Nephritis With Concurrent Anti-GBM and ANCA Positivity: A Rare Case Report

**DOI:** 10.1155/crin/4767868

**Published:** 2025-09-09

**Authors:** Thi Trinh Cao, Huy Thong Pham, Van Khanh Bui, Thi Mai Huong Nguyen, Van Phuong Ly, Van Dan Bui, Nguyen Hoang Thai, Minh Hoang Nguyen, Hoang Phuong Nguyen

**Affiliations:** ^1^Allergy and Clinical Immunology Department, Hanoi Medical University, Hanoi, Vietnam; ^2^Allergy and Clinical Immunology Center, Bach Mai Hospital, Hanoi, Vietnam; ^3^Department of Allergy, Immunology and Dermatology, e Hospital, Hanoi, Vietnam

## Abstract

**Background:** Rapidly progressive lupus nephritis (LN) with concurrent positivity for anti-glomerular basement membrane (anti-GBM) antibodies and antineutrophil cytoplasmic antibodies (ANCAs) represents an exceptionally rare and severe autoimmune overlap. Early identification and timely intervention are critical to prevent irreversible renal damage.

**Case Presentation:** A 23-year-old woman with systemic lupus erythematosus presented with acute kidney injury, nephrotic-range proteinuria, pancytopenia, and a SLEDAI score of 41. Serologic tests revealed high-titer anti-GBM antibodies and dual ANCA positivity (MPO and PR3) by the ELISA technique. Although the patient experienced mild hemoptysis and a significant drop in hemoglobin, MSCT of pulmonary vasculature and parenchyma did not reveal alveolar hemorrhage or vascular lesions. Due to contraindications to renal biopsy, she was empirically treated with pulse-dose corticosteroids and plasma exchange, followed by oral corticosteroids and mycophenolate mofetil. Anti-GBM antibodies became undetectable after seven sessions. The patient achieved full clinical, biochemical, and renal remission within 2 months.

**Conclusion:** This case highlights the importance of early serologic evaluation and prompt immunosuppressive therapy in rapidly progressive LN with anti-GBM/ANCA overlap, particularly when histopathological confirmation is not feasible.

## 1. Introduction

Systemic lupus erythematosus (SLE) is a chronic, multisystem autoimmune disease characterized by the production of a wide array of autoantibodies and immune complex deposition that can affect virtually any organ system. Among its many manifestations, renal involvement—termed lupus nephritis (LN)—is one of the most severe and prognostically significant complications. LN occurs in approximately 30% to 60% of patients with SLE and is a major contributor to both morbidity and mortality. The clinical spectrum of LN is broad, ranging from asymptomatic urinary abnormalities to rapidly progressive glomerulonephritis, nephrotic syndrome, and end-stage renal disease. Anti-glomerular basement membrane (anti-GBM) disease, in contrast, is a rare but fulminant form of autoimmune glomerulonephritis characterized by circulating autoantibodies targeting the noncollagenous domain of type IV collagen in the glomerular and alveolar basement membranes. While anti-GBM disease typically presents as isolated pulmonary-renal syndrome, its coexistence with SLE is exceedingly rare and has been reported only in isolated case studies. Even more unusual is the concurrence of anti-GBM antibodies and antineutrophil cytoplasmic antibodies (ANCAs) in a patient with SLE, which represents a unique and complex autoimmune overlap with poorly defined pathogenesis and prognosis. Triple seropositivity—anti-GBM, MPO-ANCA, and PR3-ANCA—in a patient with active lupus flare is extremely uncommon and may reflect a profound state of immune dysregulation, epitope spreading, or overlapping vasculitic mechanisms. These patients often present with rapidly progressive renal failure and may develop pulmonary hemorrhage requiring urgent immunosuppressive intervention.

This case report highlights the rarity of anti-GBM and ANCA positivity in a patient with active SLE. We emphasize the importance of early diagnosis and rapid immunosuppressive therapy to prevent irreversible renal injury. The overlap between anti-GBM disease, ANCA-associated vasculitis, and LN complicates the clinical presentation and management, making early serologic evaluation crucial in guiding therapy.

## 2. Case Presentation

A 23-year-old female with a one-year history of SLE, diagnosed according to the 2019 EULAR/ACR classification criteria, was admitted with high-grade fever, oral ulcers, malar rash, and joint pain, consistent with a severe disease flare. At initial diagnosis 1 year earlier, she had presented with arthritis, alopecia, malar rash, and thrombocytopenia, without clinical or laboratory evidence of renal involvement. Immunological evaluation revealed positive antinuclear antibodies (ANAs, homogeneous pattern), elevated anti-double-stranded DNA (anti-dsDNA) titers, and hypocomplementemia. Serum creatinine and urinalysis were normal, and renal biopsy was not indicated. The patient received high-dose oral prednisolone (1-2 mg/kg/day), gradually tapered to 4 mg/day, along with hydroxychloroquine (200 mg/day). She achieved clinical and immunological remission, with normalization of complement levels and anti-dsDNA titers. During regular follow-up over the preceding 3 months, laboratory tests remained stable, with no evidence of thrombocytopenia and LN.

One week before admission, the patient experienced diarrhea, abdominal pain, and fever, suggesting a possible infectious or inflammatory trigger for her current flare. Comprehensive infectious workup including dengue virus, influenza, hepatitis B, hepatitis C, and measles IgM was negative. The patient was not pregnant.

**Figure 1:**
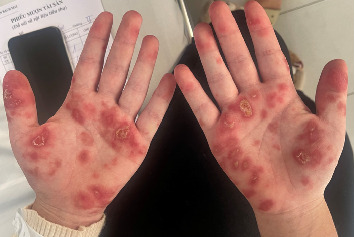
Palmar erythematous maculopapular rash with central crusted lesions before treatment.

At admission, the patient demonstrated rapid clinical deterioration with nephrotic-range proteinuria and acute kidney injury. The SLE Disease Activity Index (SLEDAI) score was 41, indicating severe disease activity. On examination, she was hypertensive and exhibited typical features of active SLE flare, including malar rash, oral ulcers, palpable vasculitic lesions (Figure 1), and bilateral lower limb edema. Her vital signs were: temperature 38.5°C, blood pressure 150/95 mmHg, heart rate 98 bpm, respiratory rate 18 breaths/min, and oxygen saturation 98% on room air.

Initial laboratory investigations revealed pancytopenia with hemoglobin 84 g/L, white blood cell count 1.46 × 10^9^/L, absolute neutrophil count 1.21 × 10^9^/L, lymphocyte count 0.2 × 10^9^/L, and platelet count 78 × 10^9^/L. A direct Coombs test was positive.

Of particular concern, serum creatinine on admission was 84 μmol/L and rose rapidly over the following days to 144 μmol/L on day 3 and 186 μmol/L by day 5, consistent with acute kidney injury. This was accompanied by a progressive decline in urine output to less than 500 mL/day. Serum albumin was low at 23 g/L, with total protein 61.5 g/L. Lipid profile showed hyperlipidemia.

Urinalysis demonstrated a urine protein-to-creatinine ratio (UPCR) of 220.7 mg/mmol, hematuria with 200 red blood cells/μL, and pyuria with 125 white blood cells/μL (Table 1).

**Figure 2:**
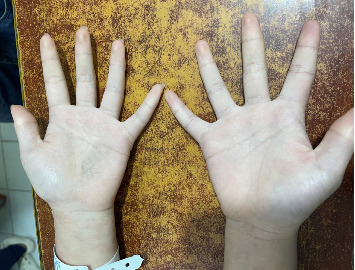
Complete resolution of palmar lesions after 2 months of immunosuppressive therapy.

Immunological testing revealed a positive ANA (homogeneous pattern 2+ by IIF), markedly elevated anti-dsDNA (> 150 IU/mL), anti-Smith antibody (98.6 U/mL), and significantly reduced complement levels (C3 0.37 g/L, C4 0.04 g/L). Serum ferritin was also elevated at 5987 ng/mL. Anti-GBM antibody was positive at 63.8 AU/mL. Both MPO-ANCA (57.8 AU/mL) and PR3-ANCA (60.0 AU/mL) were positive by the ELISA technique. Antiphospholipid antibodies were negative. Coagulation parameters were within normal limits, except for markedly elevated D-dimer at 6.819 mg/FEU. Chest X-ray and abdominal ultrasound revealed no abnormalities. On the third day of admission, prior to initiation of plasma exchange (PEX), the patient developed mild hemoptysis accompanied by a rapid decline in hemoglobin from 85 to 72 g/L within 24 h, raising suspicion for pulmonary involvement. However, multislice computed tomography (MSCT) of the pulmonary vasculature and parenchyma revealed no evidence of alveolar hemorrhage, pulmonary infiltrates, or vascular lesions. Despite clear indications for renal biopsy, the procedure could not be performed initially due to active thrombocytopenia, high bleeding risk, and rapidly worsening renal function. Given the severe disease course and triple positivity for anti-GBM, MPO-ANCA, and PR3-ANCA, immediate high-dose intravenous methylprednisolone (500 mg/day for 3 days) and daily PEX (50–60 mL/kg/session) were initiated. Anti-GBM antibodies converted to negative after seven sessions. The patient was transitioned to oral methylprednisolone (0.5–1 mg/kg/day) and maintenance therapy with mycophenolate mofetil (MMF) 2 g/day. Cyclophosphamide and rituximab were considered; however, given the patient's young age, female sex, fertility preservation concerns, and rapid favorable response to initial therapy, MMF was selected. During recovery, with improvement in platelet count, renal function, and proteinuria, renal biopsy was reconsidered. However, in light of the patient's rapid clinical and biochemical remission and following a multidisciplinary discussion, the procedure was deferred to avoid potential procedural risks. Skin biopsy was also not performed as the cutaneous lesions resolved completely with immunosuppressive therapy. After 2 months of treatment, the patient achieved complete clinical (Figure 2), biochemical, and renal remission. Hydroxychloroquine (200 mg/day) was continued throughout the disease course, as per standard lupus management. The detailed clinical course, laboratory evolution, and therapeutic timeline are summarized in Table 2.

## 3. Discussion

LN remains one of the most serious complications of SLE, affecting up to 60% of patients and contributing significantly to morbidity and mortality [1]. Immune complex deposition and complement activation within the glomeruli drive renal inflammation and damage in LN.

Triple positivity for anti-GBM antibodies, ANCAs, and SLE serology represents an exceptionally rare autoimmune overlap with complex, poorly understood pathogenesis [2]. Anti-GBM disease typically presents with rapidly progressive glomerulonephritis and pulmonary hemorrhage, whereas ANCA-associated vasculitis often leads to pauci-immune glomerulonephritis and systemic small-vessel vasculitis.

While false-positive ANCA or anti-GBM results may occur in ANA-positive patients, particularly when using ELISA-based assays, the presence of severe renal dysfunction and compatible clinical features in this patient strongly suggests true seropositivity [3]. Moreover, dual ANCA and anti-GBM positivity has been reported in both anti-GBM disease and lupus vasculitis, complicating the clinical course and prognosis [4].

This patient presented with rapidly progressive renal impairment, nephrotic-range proteinuria, pancytopenia, and high disease activity (SLEDAI 41). The development of mild hemoptysis and a significant drop in hemoglobin on day three of admission, despite unremarkable pulmonary imaging, raised further suspicion for pulmonary capillaritis in the context of anti-GBM/ANCA overlap. Coexistence of anti-GBM antibodies and ANCA positivity in SLE is associated with a more aggressive disease course and an increased risk of irreversible renal injury [5]. Early, aggressive immunosuppressive therapy combining high-dose corticosteroids, PEX, and cytotoxic or antiproliferative agents is critical to improve outcomes. The KDIGO guidelines and recent studies support the use of PEX in anti-GBM and ANCA-associated vasculitis, particularly in the presence of severe renal or pulmonary involvement [6]. Several case reports and cohort studies have highlighted the potential for renal recovery with early PEX and immunosuppressive therapy. Nasr et al. demonstrated that patients with anti-GBM and ANCA-associated vasculitis frequently experience crescentic glomerulonephritis and pulmonary hemorrhage, but timely intervention with PEX and immunosuppressants significantly improves renal outcomes [7].

Similarly, McAdoo et al. reported a case of LN with dual anti-GBM and ANCA positivity, complicated by rapidly progressive renal failure. The patient responded well to a combination of high-dose corticosteroids, PEX, and cyclophosphamide, emphasizing the need for aggressive, multimodal therapy in such complex presentations [6].

While cyclophosphamide and rituximab are considered first-line agents for ANCA-associated vasculitis and anti-GBM disease, MMF has become an established option for both induction and maintenance therapy in LN, particularly among non-White populations [8].

A recent study by Moroni et al. demonstrated that MMF significantly improved renal outcomes in patients with active LN, including those with dual positivity for ANCA and anti-GBM antibodies. In our patient, the combination of MMF, corticosteroids, and PEX contributed to stabilization of renal function and prevention of further deterioration [9].

In young female patients, MMF offers the advantage of preserving fertility. In this case, MMF was selected as maintenance therapy based on the patient's demographic profile and her rapid, favorable response to initial treatment. Renal biopsy could not be performed during the acute phase due to thrombocytopenia and a high risk of bleeding. Although reevaluated during the recovery phase, the procedure was ultimately deferred in light of complete clinical and serological remission. Similarly, skin biopsy was not pursued, as the cutaneous lesions resolved fully with immunosuppressive therapy.

The favorable renal outcome observed in this patient may be attributed to early recognition and timely initiation of aggressive immunosuppression including high-dose corticosteroids and PEX.

Ongoing monitoring includes monthly clinical evaluation, serum creatinine, urinalysis, complement levels, anti-dsDNA, and serial measurements of ANCA and anti-GBM antibody titers. Maintenance therapy with MMF and low-dose corticosteroids is planned for a minimum of 12 months.

This case underscores the importance of early serological assessment and prompt immunosuppressive intervention in patients presenting with rapidly progressive LN and concurrent anti-GBM/ANCA positivity. In the absence of histopathological confirmation, individualized, multimodal therapy remains essential to achieving favorable renal and systemic outcomes in this rare overlap syndrome (Table 3).

## 4. Conclusion

This report highlights a rare and complex presentation of rapidly progressive LN with concurrent anti-GBM and ANCA positivity. The favorable clinical outcome, despite the absence of histopathological confirmation, emphasizes the importance of early serological evaluation and timely, aggressive immunosuppressive treatment. Given the potential for severe renal and pulmonary involvement in such overlap syndromes, clinicians should maintain a high index of suspicion in atypical or rapidly progressive SLE flares and initiate prompt, individualized, multimodal therapy to optimize patient outcomes.

## Figures and Tables

**Table 1:** Laboratory parameters before and after treatment.

Parameter	Reference range	Before treatment (lowest)	After 2 months
Hemoglobin (g/L)	120–155	84	120
WBC (G/L)	4–10	1.46	5.6
Platelet (G/L)	150–400	78	210
Creatinine (μmol/L)	45–84	186	48
Albumin (g/L)	35–52	24.5	38.6
C3 (g/L)	0.9–1.8	0.37	0.96
C4 (g/L)	0.1–0.4	0.04	0.16
Anti-dsDNA (U/mL)	Negative (< 25)	> 150	100.1
Anti-Sm (U/mL)	Negative (< 15)	96.8	23.8
Anti-GBM antibody (AU/mL)	Negative(< 12)	63.8	Negative
MPO-ANCA (AU/mL)	Negative (< 12)	57.8	Negative
PR3-ANCA (AU/mL)	Negative (< 12)	60.0 U/mL	Negative
UPCR (mg/mmol)	< 50	220.7	24
Urine ERY cells (μL)	Negative	200	Negative
LEU cells (μL)	Negative	125	Negative

**Table 2:** Clinical and laboratory course summary.

Day	Clinical findings	Laboratory findings	Treatment
Day 0 (admission)	High-grade fever, malar rash, oral ulcers, limb edema, BP 150/95 mmHg. SLEDAI = 41	Creatinine 84 μmol/L, Hb 84 g/L, WBC 1.46 × 10^9^/L, platelets 78 × 10^9^/L, UPCR 220.7 mg/mmol, C3 0.37 g/L, C4 0.04 g/L, anti-GBM 63.8 AU/mL, MPO/PR3 ANCA positive	Supportive care, evaluation. Biopsy deferred due to thrombocytopenia
Days 1–3	Rapidly rising creatinine (84 ⟶ 186 μmol/L), thrombocytopenia (78 G/L), hemoptysis, Hb drop (85 ⟶ 72 g/L)	Creatinine rose to 186 μmol/L, Hb dropped to 72 g/L, platelets remained low, D-dimer 6.819 mg/FEU	High-dose IV methylprednisolone (500 mg/day × 3 days) + daily plasma exchange started
Days 3–10	Progressive renal failure, oliguria	Ongoing rise in creatinine, oliguria < 500 mL/day, persistent high proteinuria	Continuation of PLEX, corticosteroids
Post-plasma exchange (day 10)	Resolution of hemoptysis, improvement in urine output and general condition	Anti-GBM turned negative after 7 PLEX sessions. Improved labs	Transition to oral corticosteroids + MMF 2 g/day
Weeks 2–4	Rash resolved, platelet count improved, urine protein decreased, hematuria resolved	Improved creatinine, UPCR declined significantly, platelets normalized	Maintenance with MMF + low-dose corticosteroids. HCQ continued
Week 8	Complete clinical remission, disappearance of rash, normalization of renal function and urinalysis	Normal serum creatinine, urinalysis, UPCR, and complements. ANCA and anti-GBM remain negative	Long-term follow-up with MMF, low-dose corticosteroids, and HCQ

**Table 3:** Summary of similar reported cases from the literature.

Author (year)	Diagnosis	Clinical features	Treatment	Outcome
McAdoo et al. (2017)	LN with anti-GBM and ANCA positivity	Rapidly progressive renal failure, dual positivity	Steroids, PEX, cyclophosphamide	Significant renal recovery
Sharmeen et al. (2018)	LN + ANCA + overlap	Pauci-immune GN, refractory to standard therapy	Steroids, PEX, MMF, rituximab	Partial response

*Note:* GN: glomerulonephritis; PEX: plasma exchange; MMF: mycophenolate mofetil.

Abbreviation: LN, lupus nephritis.

## Data Availability

Data sharing is not applicable to this article as no datasets were generated or analyzed during the current study.
